# Immediate vs. Delayed Placement of Immediately Provisionalized Self-Tapping Implants: A Non-Randomized Controlled Clinical Trial with 1 Year of Follow-Up

**DOI:** 10.3390/jcm12020489

**Published:** 2023-01-06

**Authors:** Paolo Carosi, Claudia Lorenzi, Riccardo Di Gianfilippo, Piero Papi, Andrea Laureti, Hom-Lay Wang, Claudio Arcuri

**Affiliations:** 1 Department of Chemical Science and Technologies, PhD in Materials for Health, Environment and Energy–Dentistry, University of Rome Tor Vergata, 00133 Rome, Italy; 2 Department of Periodontics and Oral Medicine, School of Dentistry, University of Michigan, Ann Arbor, MI 48109, USA; 3 Department of Oral and Maxillo-Facial Sciences, Sapienza University of Rome, 00185 Rome, Italy; 4 Department of Clinical Sciences and Translational Medicine, School of Dentistry, University of Tor Vergata, 00133 Rome, Italy

**Keywords:** computer-guided surgery, immediate loading, immediate placement, immediate temporization, primary stability, single implant

## Abstract

This study aimed to examine the clinical and esthetic outcomes of immediately provisionalized self-tapping implants placed in extraction sockets or healed edentulous ridges one year after treatment. Sixty patients in need of a single implant-supported restoration were treated with self-tapping implants (Straumann BLX) and immediate provisionalization. The implant stability quotient (ISQ) and insertion torque were recorded intraoperatively. After one year in function, the implant and prosthesis survival rate, pink esthetic score (PES), white esthetic score (WES), and marginal bone levels (MBL) were assessed. Sixty patients received 60 self-tapping implants. A total of 37 implants were placed in extraction sockets and 23 in edentulous ridges, and then all implants were immediately provisionalized. All implants achieved a high implant stability with a mean insertion torque and ISQ value of 58.1 ± 14.1 Ncm and 73.6 ± 8.1 Ncm, respectively. No significant differences were found between healed vs. post-extractive sockets (*p* = 0.716 and *p* = 0.875), or between flap vs. flapless approaches (*p* = 0.862 and *p* = 0.228) with regards to the insertion torque and ISQ value. Nonetheless, higher insertion torque values and ISQs were recorded for mandibular implants (maxilla vs. mandible, insertion torque: 55.30 + 11.25 Ncm vs. 62.41 + 17.01 Ncm, *p* = 0.057; ISQ: 72.05 + 8.27 vs. 76.08 + 7.37, *p* = 0.058). One implant did not osseointegrate, resulting in an implant survival rate of 98.3%. All implants achieved PES and WES scores higher than 12 at the 1-year follow-up. The clinical use of newly designed self-tapping implants with immediate temporization was safe and predictable. The implants achieved a good primary stability, high implant survival rate, and favorable radiographic and esthetic outcomes, regardless of the immediate or delayed placement protocols.

## 1. Introduction

The rehabilitation of partially or completely edentulous jaws with dental implants is a preferred treatment approach these days [1,2,3,4]. Different surgical and prosthetic protocols have been proposed to optimize both implant success and patient satisfaction [5,6,7]. Among them, immediate implant placement and immediate provisionalization were favorably preferred by patients and clinicians because of the reduced treatment time [8,9]. However, both immediate placement and immediate provisionalization are technique-sensitive and may result in a higher chance of implant failure and a greater risk of mucosal recession [10,11] when compared to their delayed counterparts. To allow for immediate provisionalization, one of the prerequisites is implant primary stability, which is often represented by an insertion torque >30 Ncm and an implant stability quotient (ISQ) value ≥ 60 [12,13]. A high insertion torque value could be a good indicator for immediate loading, especially when implants are placed in extraction sockets or compromised ridge dimensions [12,13]. However, a high implant primary stability is not always possible to achieve and is often influenced by anatomical and surgical factors such as the bone density, the local anatomy, the drilling protocol, or the implant macrodesign [14]. Furthermore, excessive torque might trigger a bone micro-fracture and bone necrosis that could lead to the uncontrolled loss of crestal bone [15,16]. Despite the advancement made by the implant industry over the decades, research is still needed to design the ideal implant macrodesign that is able to predictably achieve a high insertion torque without causing excessive stress on the cortical bone. The newly designed investigated implants, featured by a self-cutting and self-tapping thread design, a tapered condensing body, a core made of a titanium–zirconium alloy (Roxolid^®^), and a sandblasted acid-etched surface, were released with the goal of improving the predictability of the insertion torque in a large range of clinical presentations. Preclinical [17,18] animal studies have investigated the torque-related behavior of the investigated implants and have always reported that favorable primary stability is achieved with this novel macrodesign. However, to the best of our knowledge, no case series was ever published to report on human data in a variety of clinical scenarios. Therefore, it was the objective of the present case series to investigate human clinical data on the use of newly designed implants, with a focus on primary stability and the 1-year radiographic and clinical outcomes of immediately provisionalized implants.

## 2. Materials and Methods

### 2.1. Study Design

This study fulfilled the Declaration of Helsinki as revised in 2013 and was reported in compliance with the STROBE guidelines [19]. The protocol was revised and approved by the ethical committee of “La Sapienza University of Rome” with identification 0000213 and registered on isRCTn.com with protocol number ISRCTN76538898. Patients in need of a single implant-supported fixed dental prosthesis (FDP) in the anterior area of either jaw were invited to participate. Interested participants were informed of the nature of the study, benefits, risks, and possible alternative treatments and provided signed consent prior to inclusion in the study. Patients were enrolled from September to December 2018, treated between January and November 2019 by two expert surgeons (P.C. and P.P.), and received follow-up in a single dental clinic for 1 year after the prosthetic function.

#### Inclusion/Exclusion Criteria

**Figure 1:**
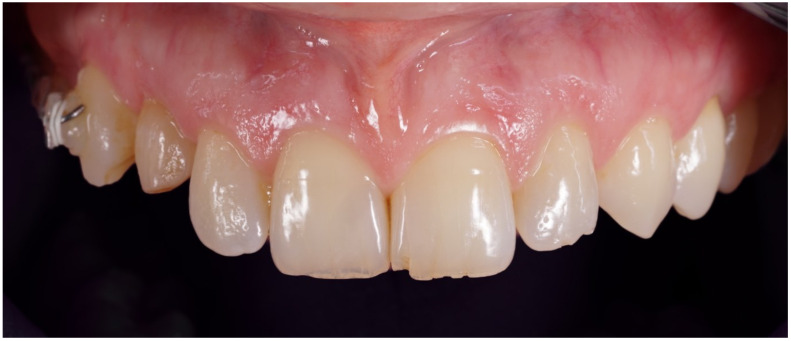
Preoperative frontal view.

**Figure 2:**
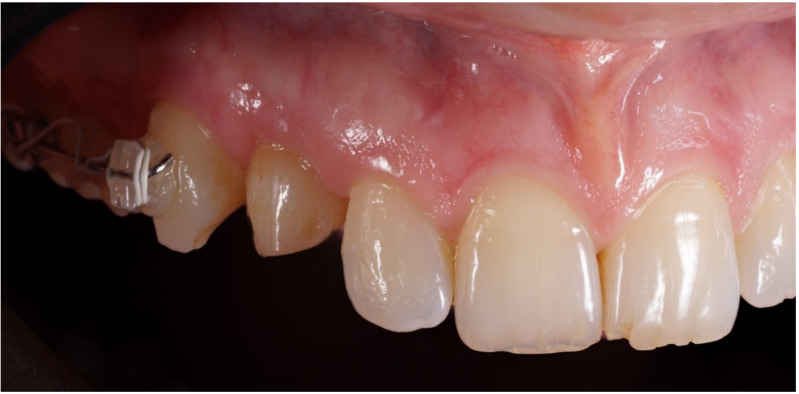
Lateral preoperative view.

**Figure 3:**
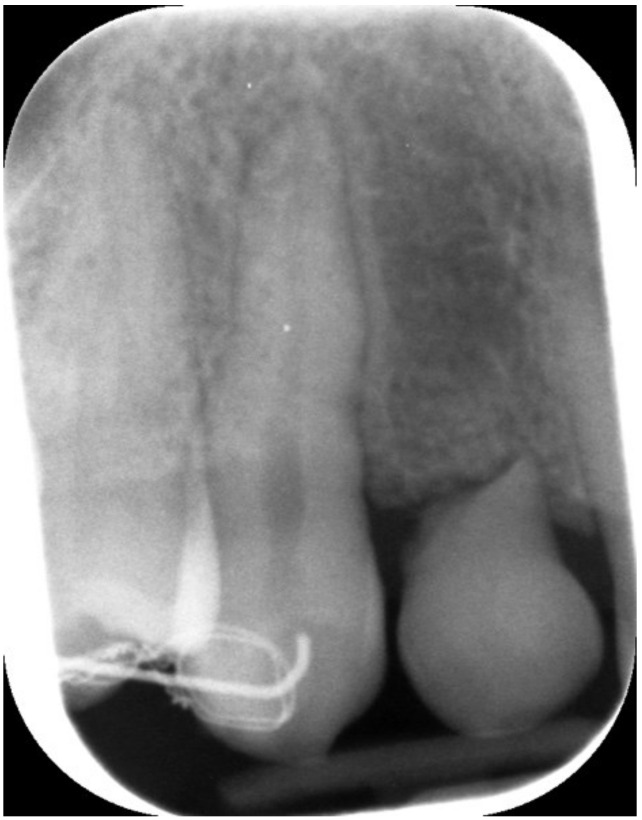
Periapical X-ray showing deciduous canine.

Patients who met the following inclusion criteria were invited to participate: >18 years old, systemic health recorded as an American Society of Anesthesiologists (ASA) I or II [20], good oral hygiene (full-mouth bleeding and plaque indices ≤25%), one permanent missing tooth or failing tooth in the anterior zone of either arch, a patient desire to have a single implant-supported fixed dental prosthesis to replace this missing tooth, an intact facial socket wall after the extraction, and a favorable bone quantity for placement of an implant with a diameter of at least 3.5 mm and a length of at least 10 mm (Figure 1, Figure 2 and Figure 3).

Patients were excluded from the study if they presented with: a systemic contraindication for oral surgical procedures; ASA III or IV; pregnancy or nursing during the study period; a history of steroid or bisphosphonate medication use; a history of alcohol or drug abuse; current heavy smoking behavior (≥10 cigarettes/day); radiation therapy to the head or neck region within the last 5 years; untreated periodontitis; the absence of opposing teeth; or the unavailability to attend regular follow-up visits.

### 2.2. Surgical Protocol

Before the surgery, a cone beam computed tomography (CBCT) scan (Orthophos XG 3D, Dentsply-Sirona, York, PA, USA) [21,22], an intraoral scan (Trios 3, 3Shape, Copenhagen, Denmark), or the digitization (D2000, 3Shape, Copenhagen, Denmark) of a model cast were merged and analyzed for a digital prosthetic treatment plan using implant planning software (coDiagnostix, Dental Wings, Montreal, Canada) [23]. A surgical guide was printed accordingly and checked intraorally before surgical intervention. Implant osteotomies were prepared following the manufacturer’s guidelines using a dedicated drill set for computer-assisted implant placement (Straumann AG, Basel, Switzerland). Low-speed drilling (40 rotations per minute (RPM)) was performed from the second to the last drill in order to avoid bone overheating and to collect autologous bone for possible bone graft materials [24]. A fully tapered implant with a self-cutting and self-tapping design, a condensing body, a Roxolid^®^ core, and a SLActive^®^ surface (BoneLevel X, Straumann AG, Basel, Switzerland) was placed by two experienced clinicians (P.C. and P.P.) in an ideal three-dimensional position by following the prosthetic digital design [25]. If any site required bone augmentation, the mucoperiosteal flaps were raised and guided bone regeneration (GBR) was performed with a 50–50% combination of deproteinized bovine bone (Bio-Oss^®^, Geistlich Pharma, Wolhusen, Switzerland) and autogenous bone, covered with an absorbable collagen membrane (Bio-Guide^®^, Geistlich Pharma AG; Fibrogide, Geistlich Pharma, Delhi, India) [26]. After the implant placement, the surgical template was removed, the implant insertion torque was measured, and the ISQ was recorded using a patented resonance frequency analysis (RFA) technology (Osstell, W&H, Göteborg, Sweden). A screw-retained temporary abutment was secured up to 15 Ncm onto the implant. Single-interrupted, internal mattress, or sling polyglycolic acid (PGA) sutures were used to approximate the flaps (Vicryl, Ethicon, Somerville, NJ, USA). Patients were instructed to rinse twice a day with 0.2% chlorhexidine mouthwash, to have a soft diet for one month, and to avoid any trauma to the surgical sites.

### 2.3. Prosthetic Protocol

**Figure 4:**
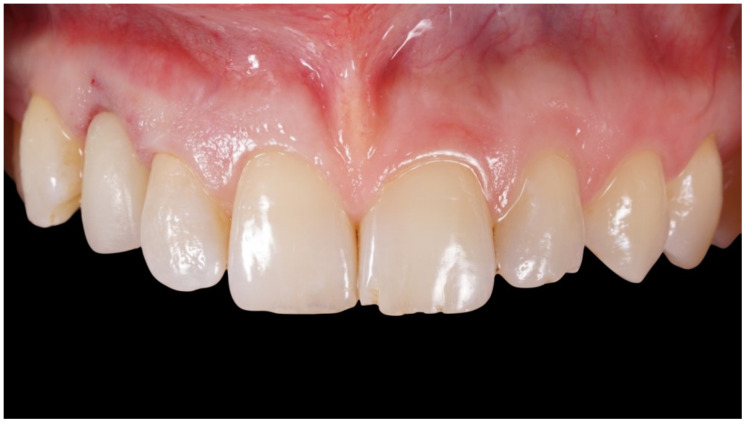
Immediate temporization of immediately placed implant.

**Figure 5:**
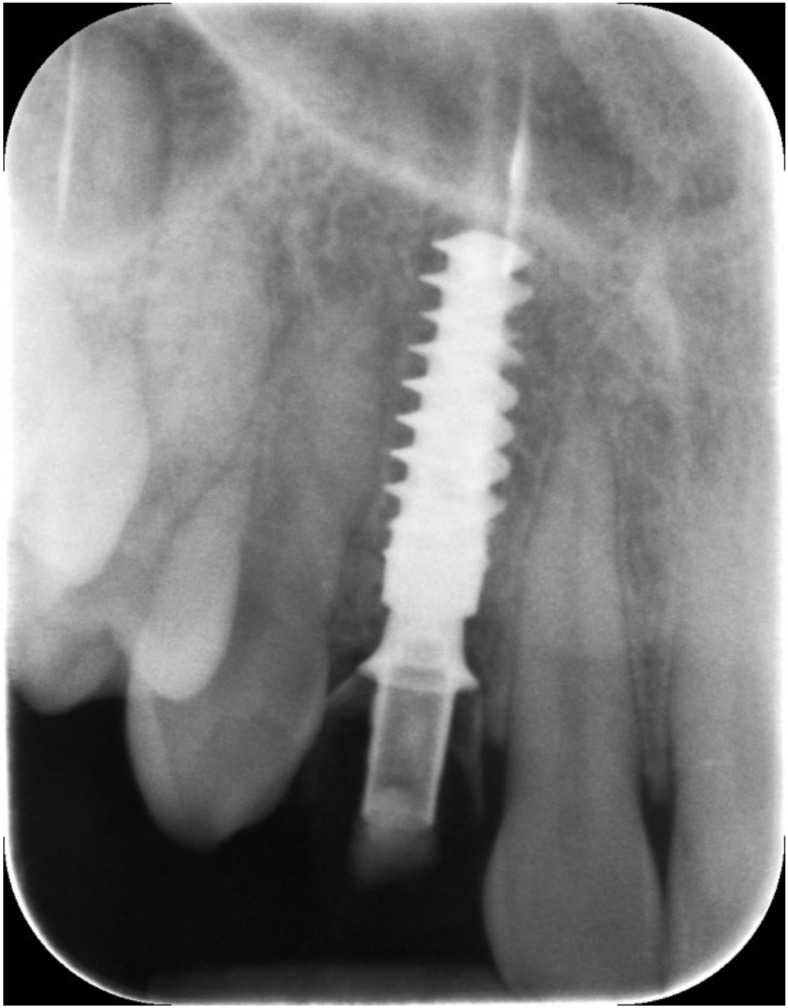
Periapical X-ray at immediate temporization.

A screw-retained interim prosthesis was designed based on the digital plan and produced from a block of polymethyl methacrylate. The abutment was merged to the temporary crown intraorally with a self-curing resin and then finished and polished (Figure 4 and Figure 5).

**Figure 6:**
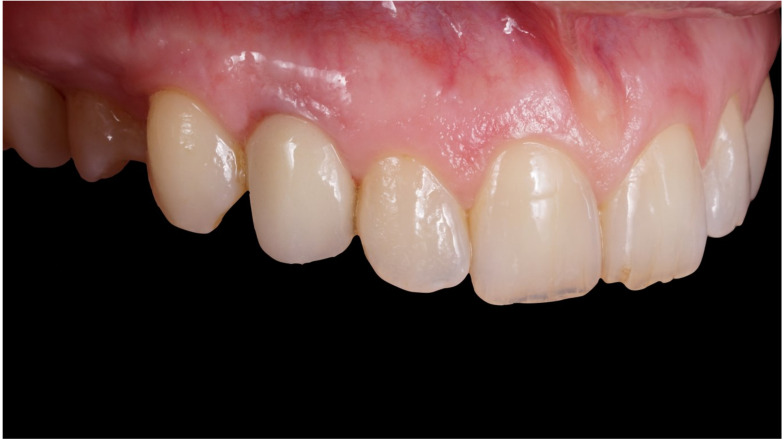
Healing of soft tissues around the temporary crown.

**Figure 7:**
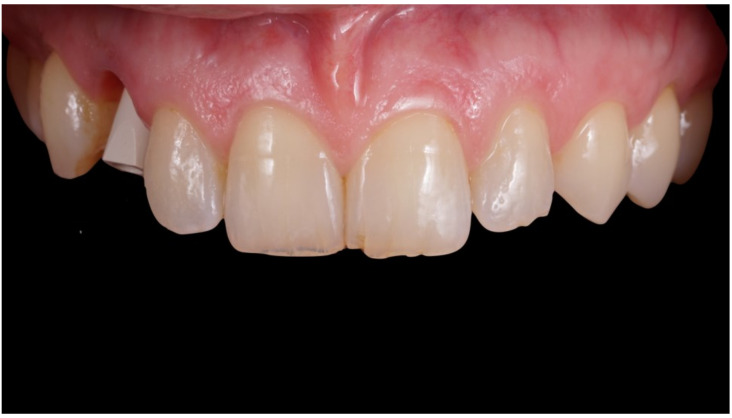
Scan abutment in position to record intraoral scan.

The occlusion was checked to avoid any contact. Patients were re-evaluated after 7–10 days to check the occlusion again, if suture removal was required, and to deliver hygiene instructions. The healing period for the osseointegration ranged from 6 to 8 weeks in the mandible and 8 to 12 weeks in the maxilla. Thereafter, the temporary crown was removed, and implant healing was checked by screwing the temporary abutment to a 35 Ncm torque. If the implant had mobility or produced any pain, soft-tissue irritation, or suppuration, it was removed and classified as “failed”. If not, a digital impression was taken by means of an IOS and dedicated scan-bodies to record the soft-tissue sculpturing and implant coordinates (Figure 6 and Figure 7).

**Figure 8:**
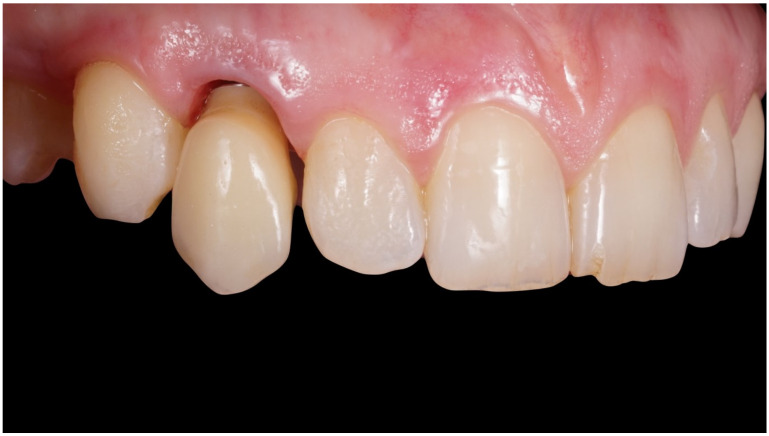
Delivery of final crown.

**Figure 9:**
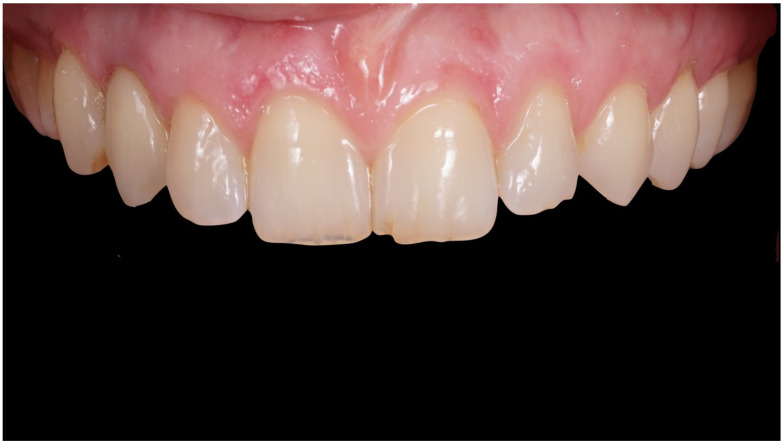
Definitive crown after 1 year of follow-up, frontal view.

**Figure 10:**
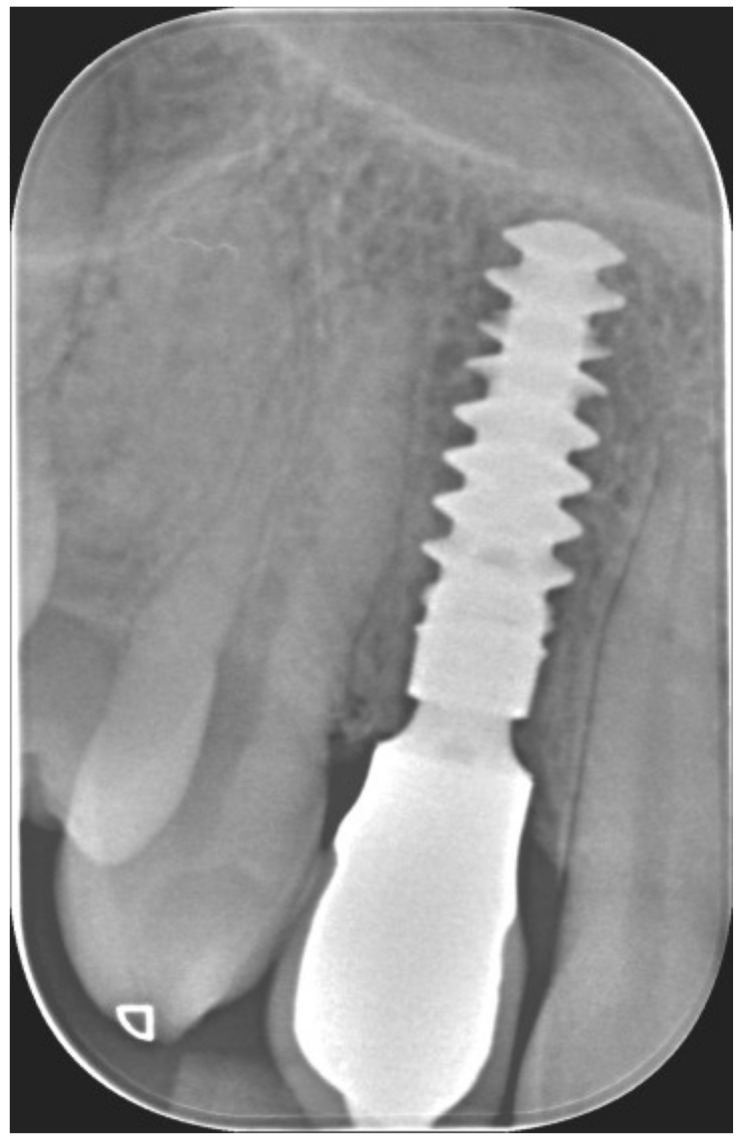
Periapical X-ray at 1 year of follow-up.

The final prostheses were screw-retained lithium disilicate fused on zirconia custom abutments. The definitive crown was screwed to 35 Ncm and the screw channel was filled with composite resin (Figure 8, Figure 9 and Figure 10).

### 2.4. Follow-Up and Outcome Variables

Patients were followed up with an 8-week interval for professional maintenance. At 12 months from the delivery of the final prosthesis, patients received a radiographic and clinical examination. Postoperative complications were recorded using the classification proposed by Askar et al. [27]. Bleeding on probing (BoP), the plaque score (PS), and probing-related variables were recorded at each visit. Periapical radiographs were performed to assess the radiographic peri-implant bone level. Implant survival was defined as the presence of the implant without pain or mobility at any follow-up examination [28]. Implant success was defined in the case of a probing depth (PD) < 6 mm together with the absence of bleeding or suppuration on probing, and a radiographic marginal bone level (MBL) < 1.5 mm [29]. Prosthetic success was defined as the presence of the definitive prosthesis with patient satisfaction and without complications, including and not limited to fracture/chipping, screw loosening, and prosthesis mobility. A pink esthetic scores/white esthetic scores (PES/WES) analysis was conducted 1 year after the definitive prosthesis placement by an independent examinator by following the Belser’s classification [30]. The radiographic marginal bone level (MBL) was assessed using a standardized (e.g., customized radiograph holder with a parallel long-cone technique) periapical intraoral radiograph at the implant placement, at the prosthesis placement, and 1 year after the prosthesis delivery. The MBL was calculated as the radiographic linear distance in millimeters from the implant platform to the most coronal bone-to-implant contact. Peri-implant soft tissues such as the mucosal recession (measured from the implant fixture level with a periodontal probe in millimeters) were assessed at each professional hygiene appointment. The BoP was recorded according to the Mombelli index [31]. All measurements were recorded by a masked and calibrated independent dental hygienist who was not involved in the surgical and/or prosthetic phases of this study.

### 2.5. Statistics

Considering the implant survival rate (ISR) as the primary outcome and assuming 97.8% as the response distribution [32], a sample size of 34 implants was calculated to guarantee a standard error of 0.05 for estimating the ISR (Sample Size Calculator, Raosoft, Seattle, WA, USA). Continuous variables were reported as the mean and standard deviation (SD). The potential effect of the arch (maxilla vs. mandible), type of incision (flap vs. flapless), and implant site (healed vs. post-extractive) on the insertion torque and the ISQ was evaluated with a *t*-test. A *p*-value of 0.05 was set for statistical significance. The implant was used as a unit in the statistical analysis.

## 3. Results

Sixty dental implants (BLX Regular Base, Straumann AG) were placed in sixty patients (27 male and 33 female, mean age: 58.7 years, range: 37–89 years). All implants were immediately temporized with a fixed screw-retained interim prosthesis. A total of 36 implants (60%) were placed in the maxilla and 24 (40%) in the mandible. A total of 23 implants were placed in healed sites (38.3%) while 37 implants were placed in post-extraction sockets (61.7%). A total of 22 implants were placed flapless (36.7%) while 38 were placed after a flap was raised (68.3%). Three different implant diameters were used: 4.5 mm (61.7%), 4.0 mm (13.3%), and 3.75 mm (25%). The most used implant length was 14 mm (65%), followed by 12 mm (31.7%) and 16 mm (3.3%).

One implant out of sixty did not reach osseointegration and was classified as “failed”, accounting for an ISR of 98.3%. The failed implant was a 3.75 mm Ø × 14 mm length positioned in a healed site. The implant was removed, a flap was raised to access the site, granulation tissue was removed with curettes, and the defect was grafted with deproteinized bovine bone mineral (Bio-Oss^®^, Geistlich Pharma) and xenogeneic absorbable collagen matrix (Fibrogide, Geistlich Pharma). The healing was uneventful and, after 4 months, a new implant was placed. No other complications were recorded for the failed case. The patient who experienced implant failure was removed from the study; therefore, the statistical analysis for the outcome variables was undertaken on 59 implants.

The values of the insertion torque, ISQ, and MBL for the full sample and for the subgroup categories were reported in Table 1.

**Figure 11:**
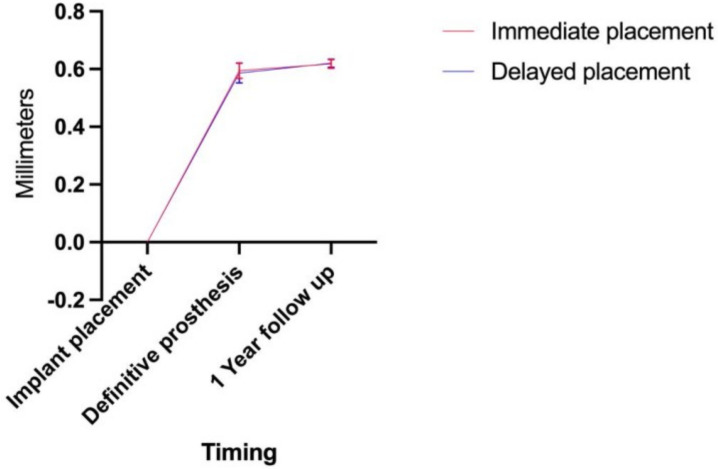
Occurrence of radiographic bone remodeling during implant healing and over 1 year of follow-up (mean ± standard deviation).

The overall insertion torque averaged 58.1 ± 14.1 Ncm. Insertion torques of 58.9 ± 13.8 Ncm and 59 ± 14.8 Ncm were achieved for post-extraction and for healed sites. The overall ISQ values averaged 73.6 ± 8.1. The ISQs for post-extraction and for healed sites were 72.3 ± 8.1 and 87.5 ± 6.8, respectively. No statistically significant differences were found for the insertion torque values and ISQ values in healed vs. post-extraction sites (*p* = 0.716 and *p* = 0.875) and in flap vs. flapless sites (*p* = 0.862 and *p* = 0.228), respectively. Higher values of insertion torque and ISQ were recorded in mandibular implants than in maxillary implants. Despite that, the difference did not achieve statistically significance (maxilla vs. mandible; insertion torque: 55.30 + 11.25 Ncm vs. 62.41 + 17.01 Ncm, *p* = 0.057; ISQ: 72.05 + 8.27 vs. 76.08 + 7.37, *p* = 0.058). A sub-analysis was performed concerning the implant lengths, diameter, and tooth position. The 14 mm-length implants had 56.7 ± 15 Ncm and an ISQ of 72.4 ± 8.3. The 12 mm-length implants had 61 ± 16.7 Ncm and an ISQ of 75.2 ± 6.9. The 16 mm-length implants had 87 ± 2.8 Ncm and an ISQ of 83.5 ± 7.7. The 4.5 mm Ø implants had 61.4 ± 14.9 Ncm and an ISQ of 74.8 ± 7.9, while the 4.0 mm Ø had 53.3 ± 9.6 Ncm and an ISQ of 70.4 ± 9.4. The 3.75 mm Ø had 52.9 ± 12.5 Ncm and an ISQ of 72.5 ± 7.6. The central incisors had 48 ± 5.5 Ncm and an ISQ of 72.7 ± 5.7, while the lateral incisors had 57.4 ± 13.3 Ncm and an ISQ of 73.9 ± 9.1. The canines had 59.2 ± 21.3 Ncm and an ISQ of 72.9 ± 6.5. At 1 year of follow-up, the mean overall MBL was 0.6 ± 0.1 mm; the MBL was 0.6 ± 0.4 mm and 0.6 ± 0.2 mm for post-extraction and healed sites, without any statistical significance between them (*p* = 0.385) (Figure 11). The mean total PES/WES value for the full set of patients was 16 + 1.85 (ranging from 12 to 19) at 1 year after the prosthesis was in function.

A total of 59 definitive single screw-retained FDPs were delivered and none of them had any prosthetic complications during the entire follow-up; as result, the prosthetics had a success rate of 100%. All implants maintained a PD lower than 5 mm throughout the follow-up period. At the last examination, two implants were diagnosed with peri-implant mucositis (3.4%) and no implants were diagnosed with peri-implantitis within the study period.

## 4. Discussion

The aim of this case-series study was to evaluate the primary stability and the 1-year outcome of newly-designed self-tapping implants, placed in extraction sockets or in healed ridges and immediately provisionalized. No differences were noted between implants placed in extraction sockets or healed ridges. Furthermore, the null hypothesis that the anatomical location (maxilla vs. mandible), type of incision (flap vs. flapless), and ridge anatomy (healed vs. post-extractive) may influence the implant insertion torque and ISQ values was rejected, as no local or surgical factors seemed to affect these variables of primary stability.

The existing literature on the investigated implant design (self-cutting and self-tapping thread design, tapered condensing body, and a core made of a titanium–zirconium alloy (Roxolid^®^)) is scarce and with heterogenous methodology. Francisco et al. [33] recently reported no statistically significant differences between the submerged and transmucosal healing of implants in minipigs when marginal bone levels were assessed. Limmeechokchai et al. [34] placed these newly designed implants in a compromised in vitro model and bovine bone blocks, which showed excellent implant stability while reducing the stress on the cortical bone.

Despite the very high torque values achieved during implant insertion, which might have increased the risk of a high MBL, the noted bone remodeling was limited and comparable to the current data in the literature [17,35,36]. It is our assumption that the reduced extent of the surgical flap, platform-switching connection, and crestal microgrooves might have contributed to reducing the biological remodeling of the bone and the associated bone loss [37,38,39]. Moreover, the combination of high primary stability values and the limited bone remodeling may also be related to the design of the implants themselves. As a matter of fact, despite the diameter and the length of the implants, the most coronal part and the body of the implants were always the same in terms of the neck diameter and body design. The neck of all the positioned implants was 3.5 mm Ø, reaching 1.9 mm Ø in the apical part. In this sense, the differences between several diameters were only in the depth of the threads. It was observed that the higher values of insertion torque were achieved by the 4.5 mm Ø implants. This can be explained by the inner design of the implant, with 1 mm of pure threads that can engage the surrounding bone. Moreover, all the implants had one single prosthetic connection, enhancing the versatility during the surgery if a shift from one diameter to another was necessary.

In the present study, one implant and no prostheses failed, accounting for an ISR and PSR of 98.3% and 100%, respectively. These results are in accordance with the current literature on immediately loaded implants [40,41]. Recent systematic reviews [33] have reported an implant survival rate of 97.3–97.9% for immediately loaded single implants in the anterior zone. Our study is in support of these findings, which implies that this specific implant macrodesign might facilitate the achievement of high primary stability without compromising bone density. Nonetheless, it is also important to point out that immediate loading is a technique-sensitive approach that requires dedicated training as well as years of experience [7,42,43,44].

Digital technologies developed in the last years have enhanced aesthetic outcomes. The possibility of previewing the final prosthetic design allows the clinician to properly plan implant coordinates according to bone and soft-tissue health. Moreover, the merging of digital data representing the bone architecture, gingival shape, intraoral status, and prosthetic design plays a crucial role in planning both implant positioning and tissue regeneration. In the present study, computer-aided implant placement was performed after careful and accurate implant and prosthetic planning. Digital-implant-assisted surgery has been largely developed in the last years, including different systems such as static and dynamic computer-assisted implant placement. Static-system implant placement has been widely used from single tooth replacement to complete arch rehabilitation, providing good clinical results in terms of invasiveness and accuracy [6]. The use of a static printed template is synonymous of pre-planned implant coordinates without the possibility of visual control of the implant recipient site while the drills are in function in combination with a dedicated surgical kit with an appropriate drill set. On the other hand, dynamic navigation surgery was developed to avoid the use of static templates and to have a full visual of the surgical field. In the last years, several studies have investigated the clinical outcomes of implants placed in the aesthetic zone by means of dynamic navigation systems [7,45]. The use of both static and dynamic computer-assisted surgery enhances clinical outcomes, such as the PES/WES, MBL, and ISQ values, probably because of the careful pre-planning according to the implant recipient sites and both the bone and soft-tissue anatomy of each treated patient.

Moreover, the mechanical stability over the follow-up period of the prostheses may be related to the use of zirconia and lithium disilicate to realize definitive prostheses. As a matter of fact, in the anterior zone of both jaws, the forces on the teeth are not axial, and this may cause chipping of the ceramic or a fracture of the abutment and frameworks [46].

This trial provides valuable information regarding the interactions between primary stability (ISQ and insertion torque) and surgical/anatomical factors for the investigated macrodesign implant during immediate temporization. This study, however, was not free from limitations. Because of the case-series design, no control group was used to compare the insertion torque and ISQ between the investigated implant and another implant of a different macrodesign. In addition, this study was a one-year follow-up study, which was not long enough to draw conclusions about the sustainability of the achieved outcomes, risk of peri-implantitis, or changes in esthetics subsequent to long-term soft-tissue remodeling. Future studies are encouraged to compare this current implant design to other commercially available implants to better understand the biological differences as well as the safety of immediate temporization. In addition, long-term data are encouraged to assess the sustainability of the documented results.

## 5. Conclusions

Within the limitations of this study, it was concluded that the insertion torque and ISQ values of the newly designed implants were consistently high. They were not significantly influenced by the implant site (healed vs. post-extractive), the anatomical location (maxilla vs. mandible), or the type of incision (flap vs. flapless), and they did not correlate with peri-implant bone remodeling. Moreover, the immediate temporization of this implant macrodesign seemed to be a safe and reliable treatment approach for the rehabilitation of a single edentulism in the anterior area. However, further prospective trials that include a control group with a different implant macrodesign and longer follow-up periods are encouraged to validate the results of this pilot cohort.

## Figures and Tables

**Table 1:** Main results of primary stability values of investigated implants.

	Implant Stability Quotien (ISQ) (Mean ± SD)	Torque (Mean ± SD, Ncm)	1-Year Radiographic Marginal Bone Loss (Mean ± SD, mm)
All implants	73.6 ± 8.1	58.1 ± 14.1	0.62 ± 0.1
Immediate implants	72.3 ± 8.1	58.9 ± 13.8	0.63 ± 0.4
Delayed implants	87.5 ± 6.8	59 ± 14.8	0.61 ± 0.2
*p*-value	0.875	0.716	0.385
Maxilla	72.05 ± 8.27	55.30 ± 11.25	0.64 ± 0.6
Mandible	76.08 ± 7.37	62.41 ± 17.01	0.60 ± 0.3
*p*-value	0.058	0.057	0.284
Flap	74.63 ± 8.50	58.07 ± 14.47	0.63 ± 0.2
Flapless	72.00 ± 7.27	58.27 ± 13.90	0.61 ± 0.4

## Data Availability

The data presented in this study are available from the corresponding author upon reasonable request.
